# Identification of striatal cell assemblies suitable for reinforcement learning

**DOI:** 10.1186/1471-2202-12-S1-P228

**Published:** 2011-07-18

**Authors:** Carlos Toledo-Suárez, Man Yi Yim, Arvind Kumar, Abigail Morrison

**Affiliations:** 1 Functional Neural Circuits Group, Faculty of Biology, University of Freiburg, 79104, Germany; 2 Bernstein Center Freiburg, University of Freiburg, 79104, Germany; 3 Neurobiology and Biophysics, Faculty of Biology, University of Freiburg, 79104, Germany; 4 Dept. Computational Biology, School of Computer Science and Communication, KTH, Stockholm, 10044,Sweden

## 

Both in vivo [1] and in vitro [2] experimental data suggest that medium spiny neurons in striatum participate in the formation of sequentially firing cell assemblies, at a timescale relevant for the presumed involvement of basal ganglia in reinforcement learning. Computational models argue that such cell assemblies are a feature of a minimal network architecture of the striatum [3]. This suggests that cell assemblies can be a potential candidate for representation of the 'system states' in the framework of reinforcement learning.

Spike patterns associated with cells assemblies can be identified by clustering the spectrum of zero-lag cross-correlation between all pairs of neurons in a network [3]. Other methods based on the dimensionality reduction of the similarity matrix of the spike trains have also been used [2,4].

Here we investigate how the identification of cell assemblies is dependent on the methodology chosen, and to what extent the statistical properties of the cell assemblies make them suitable for representation of system states in the striatum during reinforcement learning.
